# ModelArchive: A Deposition Database for Computational Macromolecular Structural Models^✩^

**DOI:** 10.1016/j.jmb.2025.168996

**Published:** 2025-02-11

**Authors:** Gerardo Tauriello, Andrew M. Waterhouse, Juergen Haas, Dario Behringer, Stefan Bienert, Thomas Garello, Torsten Schwede

**Affiliations:** Biozentrum, University of Basel, Basel, Switzerland; Computational Structural Biology, SIB Swiss Institute of Bioinformatics, Basel, Switzerland

**Keywords:** ModelArchive, FAIR databases, structural biology, macromolecular structure prediction, ModelCIF

## Abstract

A wide range of applications in life science research benefit from the availability of three-dimensional structures of biological macromolecules as they provide valuable insights into their molecular function. Recent advances in structure prediction techniques have made it possible to generate high quality computational macromolecular structural models for almost all known proteins. In this context, ModelArchive (https://modelarchive.org/) serves as a deposition database for computational models, complementing the Protein Data Bank (PDB) and PDB-IHM, which require experimental data, and specialised databases such as the AlphaFold DB. ModelArchive contains over 600,000 models contributed by researchers using a variety of modelling techniques. It supports single biological macromolecules and complexes, including any combination of polymers and small molecules. Each deposited model can be referenced in manuscripts using an immutable accession code provided by ModelArchive. Depositors are required to provide a minimal set of information about the modelling process and the expected accuracy of the resulting model, enabling scientific reproducibility and maximising the potential reuse of the models. The vast majority of models in ModelArchive use the ModelCIF format which includes coordinates and metadata, allows for programmatic validation of the models, and makes the models interoperable with structures obtained from other sources such as the PDB. The ModelArchive web service provides access to the models and search queries. Model findability is also provided in external services either through APIs or by importing data from ModelArchive.

## Introduction

Proteins, DNA, and RNA are indispensable players in all biological processes, and their functions are inextricably linked to their three-dimensional structure. The determination of structures has heretofore been conducted mainly through experimental means. However, recent developments in computational methods have resulted in notable advancements in the accurate prediction of three-dimensional macromolecular structures. The most recent illustration of these advancements is the awarding of the 2024 Nobel Prize in Chemistry to protein structure prediction and specifically for the development of AlphaFold2.^1^ Computed structure models (CSMs) can nowadays be produced with sufficient accuracy to often rival those determined by experiment, and enable new possibilities for research where experimental structures are unavailable or impractical to obtain.

The growing utility of CSMs poses new challenges in terms of reproducibility and reusability. Unlike experimental structures, in the scientific literature CSMs are not always systematically deposited or annotated. This lack of standardisation hinders the validation, reuse, and integration of CSMs into downstream studies. As the scientific community increasingly uses CSMs for tasks such as protein function determination, drug design, protein engineering, and variant analysis, the need for dedicated repositories to store, share, and document these models has become apparent.

Since its establishment in 1971, the Protein Data Bank (PDB) has functioned as a unified global repository for three-dimensional structures of biological macromolecules obtained through experimental techniques, including X-ray crystallography, nuclear magnetic resonance (NMR), and, more recently, cryo-electron microscopy (EM).^2^ However, since 2006, the PDB has not archived structures determined through computational modelling,^3^ resulting in CSMs stored in undefined locations, in incompatible formats, and lacking essential metadata. In a workshop held in 2008, leading international scientists involved with structure modelling investigated the utilisation of CSMs in biomedical research^4^ and formulated a set of recommendations for the archiving and sharing of CSMs. The workshop emphasised the value of enhancing the structural coverage of protein sequences with CSMs, the necessity for data standards and best practices for the publication and dissemination of models, and the pivotal importance of providing model accuracy estimates for CSMs. Additionally, it advocated for the establishment of an archive for CSMs that could not be deposited in the PDB.

Consequently, an initial iteration of the ModelArchive was developed as part of the Protein Structure Initiative (PSI) Structural Biology Knowledgebase.^5^ The archive incorporated CSMs that had been stored in the PDB prior to 2006 and has been accepting new depositions since 2013. ModelArchive has been designed with the specific purpose of depositing structural models that are not based on experimental data. This provides a complementary resource to the PDB, which serves the same function for experimental structures, and to PDB-IHM,^6^ which is dedicated to integrative structures.^7^

In addition to the ModelArchive, other data resources have been established to facilitate large-scale access to CSMs generated through a specific automated prediction method. These include ModBase^8^ and the SWISS-MODEL Repository,^9^ which contain CSMs for millions of proteins generated by homology modelling using Modeller and SWISS-MODEL, respectively. These models are continuously updated to reflect the latest advances in the modelling methods and the availability of input data. More recently, the AlphaFold Protein Structure Database^10^ and the ESM Metagenomic Atlas^11^ have been established. The AlphaFold database contains over 200 million proteins modelled using AlphaFold2, while the ESM atlas contains 772 million proteins modelled using ESMFold. Despite their widespread use, these repositories are limited in scope. For example, the models are all monomers, they exclude either all or large parts of viral proteins, and they are limited to a maximum sequence length. In addition, they are tied to specific modelling pipelines and do not allow external deposition, limiting their scope for wider CSM integration.

The Biological Structure Model Archive (BSM-Arc)^12^ and general-purpose repositories such as Dryad, FigShare, Zenodo, and the Open Science Framework (OSF) provide researchers with alternative storage options for the sharing of CSMs. However, these platforms lack the structural metadata and standardisation required for effective reuse and interoperability of the contained CSMs, thereby reducing their potential impact.

CSM interoperability and reuse remain a topic of ongoing research. For example, the Research Data Management Kit^13,14^ provides best-practice guidelines for the administration of computational data, whereas the ModelCIF format^15^ offers a standardised data structure for the description of CSMs. The 3D-Beacons network^16^ makes a further contribution by incorporating federated queries to multiple structural model resources, thus providing a unified platform for accessing and visualising models. These initiatives demonstrate the progress made in supporting computational models, yet they underline the need for a centralised repository that allows for depositions and that adheres to the FAIR (Findable, Accessible, Interoperable, Reusable) principles for CSMs.

The ModelArchive addresses this need by providing a dedicated platform for the deposition, curation, and sharing of CSMs. By enabling FAIR access to models and emphasising metadata standardisation, ModelArchive ensures that CSMs remain accessible and reusable for future scientific endeavours. Here we describe the design, features, and current content of ModelArchive (https://modelarchive.org/), demonstrating its potential to enhance reproducibility and accelerate discoveries in the structural biology community.

## Results and Discussion

### Data deposition pipeline

**Figure 1.**
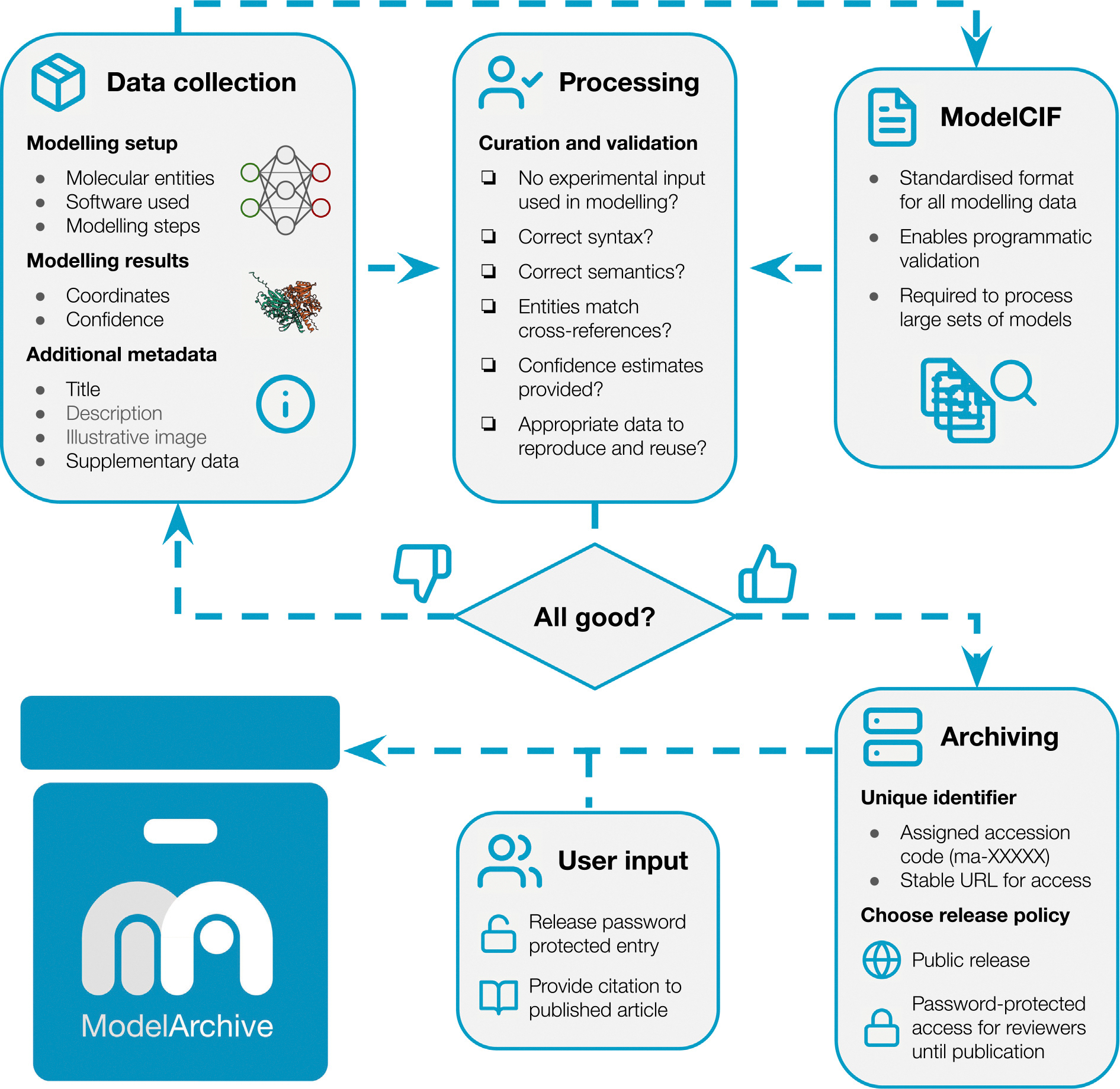
Schematic representation of the data deposition pipeline for ModelArchive, showing the iterative processing of data collected for modelling results and the archiving of an entry. Curation and validation by the ModelArchive team ensures that the provided metadata is sufficient for scientific reproducibility and has potential for reuse.

The deposition pipeline (Figure 1) for ModelArchive ensures that Computed Structure Models (CSMs) are collected, curated and archived for scientific reproducibility and reuse. The process consists of three steps. First, molecular coordinates and metadata are collected to describe the model generation process. The data is then processed, curated, and validated by the ModelArchive team. Finally, the model is securely stored with a unique accession code and made available either publicly or with restricted access for review.

ModelArchive supports the same range of molecular entities as the PDB, such as single molecules and complexes containing proteins, RNA, DNA, carbohydrates or small molecules. However, complementary to PDB or PDB-IHM, which require experimental data, ModelArchive only accepts computationally generated models where no experimental input is used in modelling. Certain types of structural data are outside the scope of ModelArchive, including molecular dynamics trajectories and protein ensembles for intrinsically disordered proteins, the latter already covered by the PED database.^17^

Deposited models in ModelArchive are categorised into three different types based on their format and metadata structure. Legacy pre-2006 models have been migrated from the PDB to preserve historical data and are stored in the legacy PDB format. Individual ModelArchive entries currently contain free text descriptions of the metadata, allowing individual models to be reproduced and evaluated while maintaining simplicity for depositors. For these entries, coordinates are stored in the standard wwPDB PDBx/mmCIF format. Ongoing efforts will ensure that these models use the ModelCIF format in the future. Large model sets already use the ModelCIF format, which is an extension of PDBx/mmCIF. This stores the metadata alongside the model coordinates in a standardised data structure. This allows for programmatic validation and the handling of large model sets, both during deposition and for further reuse of the models. Further details of the formats used for data import can be found in the supplementary material. The use of standard file formats facilitates integration with structural data from other sources. Standardised metadata ensures that deposited models can be checked for suitability for a wide range of applications in structural biology.

To ensure scientific reproducibility and maximum potential for reuse, depositors are required to provide metadata covering several essential aspects. First, the metadata must clearly describe the molecular content of the model, including descriptions of the molecular entities and cross-references to resources such as UniProtKB or NCBI’s protein database. Second, it must document the modelling process in sufficient detail, including the software used, the specific steps performed and the input data, so that others can reproduce the model. Third, submitters must provide estimates of the expected accuracy of the model. Modern modelling methods are capable not only of generating accurate structures, but also of reliably assessing their own accuracy, both globally and locally, to identify potential inaccuracies in regions of interest. Typically, the accuracy estimates provided predict the expected similarity to the unknown correct structure according to metrics such as LDDT,^18^ TM-score^19^ or DockQ.^20^ In addition, depositors provide information to improve the usability and discoverability of their models. This includes a concise title, a description of the purpose of the model, the type of model (e.g. homology-based or *de novo*), an illustrative image, the list of authors, optional funding information to acknowledge supporting grants, and an optional zip file containing supplementary data.

The ModelArchive team validates each deposition to ensure that it meets both syntactic and semantic standards. Syntactic validation confirms that all required data is included and correctly formatted, while semantic validation ensures consistency between the metadata and the model itself, for example by checking the match between the modelled sequence and its stated source. If discrepancies or missing information are identified, the submission is returned to the submitter with feedback for correction. While model confidence is visible as part of the metadata, it is not used as a criterion for rejection. This approach ensures that the repository maintains high standards without excluding potentially useful models.

Each model deposited in ModelArchive is assigned a unique accession code, such as “ma-jd-viral-22025”, which resolves to a stable URL for citation in publications (e.g. https://www.modelarchive.org/doi/10.5452/ma-jd-viral-22025). Depositors can choose to make their models publicly available immediately, or to delay public release until after publication, while providing password-protected access for peer review. Once a model has been accepted, depositors can request its release and provide citation details to link the model to the publication for which it was created. To ensure that models do not remain private or uncited, we send annual reminders to all depositors with such entries. The latest information on the deposition process can be found on the ModelArchive help page (https://modelarchive.org/help).

### Overview of current content

By the end of 2024, there were 618,491 models publicly available in ModelArchive. Each model has a unique accession code and the vast majority of models (615,828) are grouped into model sets using the ModelCIF format and have been added in the last four years. The depositions come from 279 depositors from 43 different countries. With the exception of pre-2006 legacy models migrated from the PDB, all models have been deposited by the creators of the models. Table 1 lists the current content, including each grouped model set. Model sets have a single accession code that represents the entire set (e.g. ma-jd-viral) and acts as a prefix to the accession code of each individual model (e.g. ma-jd-viral-22025). Historical growth of ModelArchive can be found in the supplementary material.

The oldest models available (ma-c1ewu, ma-cdw44, ma-ceo7o, ma-cwc6z) are from a 1978 paper that generated homology models of relaxin based on a known insulin structure.^39^ Today, most depositions are *de novo* models generated with a variant of AlphaFold. The first set of models based on ModelCIF (ma-bak-cepc) was added in late 2021 for a study in which 1,106 core eukaryotic binary complexes were identified and modelled with AlphaFold2.^21^ The complexes have been deposited in ModelArchive to make them available to the wider community, as the structures could provide insights into the biological function of proteins involved in key processes of eukaryotic cells. As a result, we observe a number of studies that have used these models.^40–42^

The models in ModelArchive are complementary to the AlphaFold database by including protein–protein complexes (ma-bak-cepc, ma-tbvar3d, ma-t3vr3, ma-low-csi, ma-rap-bacsu, ma-kul-lams, ma-dm-prc, ma-dm-hisrep, ma-osf-ppp2r2a), protein–ligand complexes (ma-tbvar3d), viral proteins (ma-asfv-asfvg, ma-jd-viral) and relevant subsets of long proteins (ma-kul-lams). Furthermore, UniProt and therefore the AlphaFold database do not contain all known or possible proteins, and some isoforms, organisms, results of metagenomics studies and designed proteins may be missing. ModelArchive contains models of representatives of metagenomic protein families (ma-nmpfamsdb, ma-fesnov), designed proteins (ma-osf-ppp2r2a) and other proteins missing in UniProt (ma-coffe-slac, ma-ornl-sphdiv, ma-tur-clump). In addition, models can be deposited to support the evaluation of modelling methods, as has been done for the benchmarking of AlphaFold2 on outer membrane beta-barrels (ma-ombbaf2) and for the benchmarking of AlphaLink (ma-rap-alink).

An advantage of ModelArchive and ModelCIF is that it can be used to describe modified modelling pipelines, including those with manual intervention, while still maintaining an interoperable set of results. For example, for a study of viral proteins,^35^ models of 67,715 eukaryotic viral proteins were deposited (ma-jd-viral). These were generated with ColabFold^43^ using MMseqs2^44^ to obtain multiple sequence alignments (MSAs), which were used as input for AlphaFold2.^1^ In addition, a custom search database based on viral proteins in RefSeq was used for most models to improve the MSAs. These aspects can be captured in ModelCIF while maintaining the same type of output and model confidence estimates (i.e. pLDDT, pTM and PAE) as for other models generated with AlphaFold2.

### Data distribution

The ModelArchive website (https://modelarchive.org/) acts as the entry point for depositing, browsing and accessing CSMs. Several indexing technologies are used to search and retrieve metadata about them. The primary database is PostgreSQL, which supports accession code lookups and simple text queries for ModelArchive users, as well as searches initiated by the Swiss Bioinformatics Resource Portal, Expasy.^45^ Once a ModelArchive entry is cited in a journal, we register its Digital Object Identifier (DOI) to make it accessible as https://doi.org/10.5452/ma-xxxxxx. Links between ModelArchive entries and journal articles are also tracked as external links in Europe PMC.

The Python web framework Django sits in front of the database, handling authentication for accessing released and unreleased depositions, and managing user and admin accounts throughout the deposition pipeline. Static content, including items requiring authorisation, is served by Nginx. Django’s REST framework provides metadata for editing and viewing entries. ModelArchive’s architecture was designed to accommodate updates to its front-end framework. It currently uses the flexible and powerful component-based JavaScript library, React.

**Figure 2.**
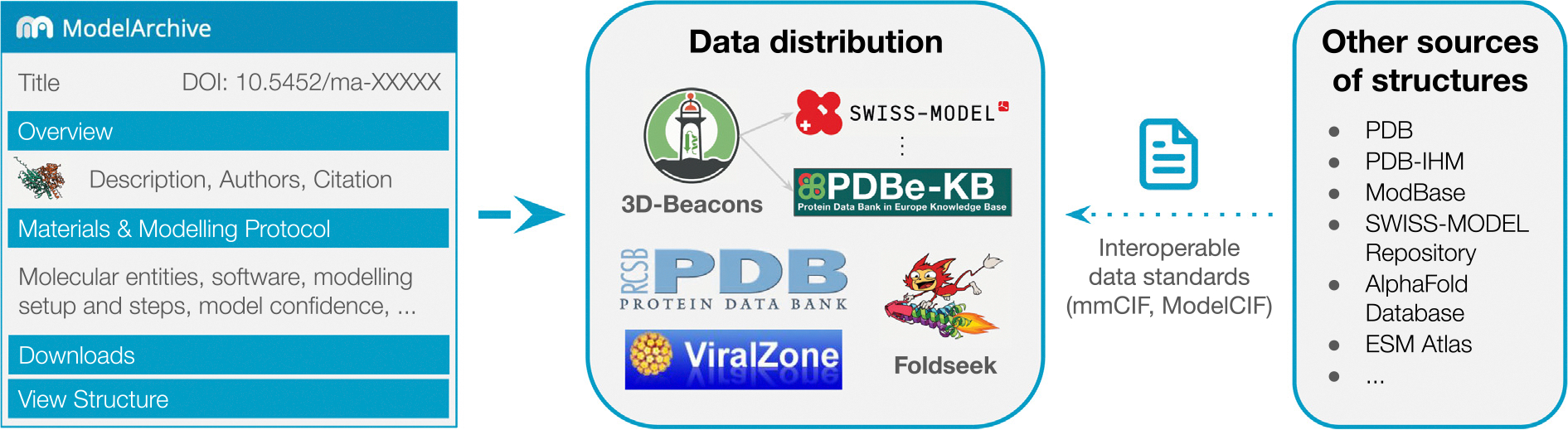
Data distribution showing how entries in ModelArchive are also made available in external services. Interoperable data standards ensure that the models can be readily integrated with structural data from other sources, including experimental structures and computed structure models.

Data in ModelArchive is distributed to external services (Figure 2), improving visibility and discoverability. It also allows integration with experimental structures and CSMs from other sources thanks to the interoperable data standards. For entries in ModelArchive linked to UniProtKB, metadata and structural coordinates can be retrieved using the 3D-Beacons API.^16^ This API is used, for example, in the SWISS-MODEL Repository^9^ and the PDBe-KB.^46^ The RCSB PDB includes selected ModelArchive models with metadata extracted from ModelCIF. ViralZone links to ModelArchive for viral proteins with available CSMs.^47^ Foldseek includes ModelArchive models in the BFMD database for structure similarity searches.^48^ These external services provide additional query capabilities which are particularly useful when CSMs are queried, accessed and combined with experimental data, as is the case in the SWISS-MODEL Repository, PDBe-KB, the RCSB PDB and Foldseek. Further architectural details can be found in the supplementary material.

By default, ModelArchive models are available under a permissive licence. Some methods, like AlphaFold 3,^49^ do not allow this due to restrictive terms of use. We allow such models to be deposited in ModelArchive with the specific terms of use explicitly displayed, but they cannot be accessed via APIs requiring a more permissive licence.

## Conclusion and Perspectives

Through its rigorous yet flexible deposition pipeline, ModelArchive provides a robust platform for the preservation and sharing of computationally generated structural models that are not based on experimental data. By adhering to the FAIR principles and emphasising metadata quality, it enables researchers to effectively access, evaluate and reuse these models. By using standardised metadata and formats, ModelArchive enables the seamless integration of computational models into existing research workflows, complementing experimental data.

ModelArchive is a major contributor and user of the ModelCIF format. The ModelArchive team has been actively assisting depositors to convert their data into valid sets of ModelCIF files. In parallel with the existing tool and library support for ModelCIF,^15^ we are now working on a data harvesting system that will be compatible with PDB-IHM,^6^ which will allow depositors to interactively generate valid ModelCIF files for individual ModelArchive entries. The ModelCIF format currently covers macromolecular structure predictions. We are working to extend the format to cover more specific details of other modelling applications, including predicted protein–ligand interactions, macromolecular complexes, predictions of different protein conformational states and results of protein design studies.

It will remain a challenge for ModelArchive to keep up with the rapid developments in the field. As the prediction of individual protein chains is already generally very accurate, the focus is shifting to assessing the accuracy of a model in functionally critical regions, e.g. interaction sites with other molecules, as this is relevant for many applications. This in turn requires new metrics for assessing the accuracy and reliability of predictions to be incorporated into ModelCIF and ModelArchive.

Widespread adoption of ModelArchive will require outreach to promote best practices and encourage CSM deposition and we will work with publishers and funding agencies to establish appropriate guidelines. Advances in AI, such as AlphaFold, are making CSMs increasingly accurate and transformative for applications such as protein function determination, drug design, protein engineering and variant analysis. By providing a platform for accessible and reusable models, ModelArchive amplifies the impact of these advances. As computational modelling continues to evolve, ModelArchive will remain critical to fostering reproducibility, collaboration and innovation, ensuring that these models drive progress in both basic and applied life sciences.

## Supplementary Material

1

## Figures and Tables

**Table 1:** Publicly visible content in ModelArchive by the end of 2024, including pre-2006 legacy models, individual non-ModelCIF ModelArchive entries, and all model sets with ModelCIF formatted files identified by their ModelArchive accession code listed in chronological order.

Type	Models	Description

pre-2006	1,373	Legacy models migrated from the PDB
non-ModelCIF	1,290	Individual entries with free-text metadata descriptions
Identifier	Models	Description

ma-bak-cepc	1,106	Yeast protein dimers^21^
ma-coffe-slac	41,932	Freshwater sponge proteins^22^
ma-tbvar3d	19	*M. tuberculosis* complexes related to antibiotic resistance
ma-ornl-sphdiv	25,134	Peat moss (Sphagnum) proteins^23^
ma-asfv-asfvg	197	African swine fever virus proteins^24^
ma-t3vr3	957	Human cancer related protein dimers^25^
ma-low-csi	929	Human protein dimers^26^
ma-ombbaf2	441	OMBB models for AlphaFold2 benchmarking^27^
ma-rap-bacsu	167	*Bacillus subtilis* dimer and trimer complexes^28^
ma-rap-alink	1,510	Models for AlphaLink benchmarking^29^
ma-tur-clump	4,224	Human isoforms from RefSeq to assess variants^30^
ma-kul-lams	55	LN-lamininopathy proteins and complexes^31^
ma-saps	21	Phytoplasma effector models^32^
ma-nmpfamsdb	80,585	Novel metagenome protein families^33^
ma-fesnov	389,522	Functionally & evolutionarily significant novel gene families^34^
ma-jd-viral	67,715	Eukaryotic virus proteins^35^
ma-dm-prc	742	Structural prediction screen for PRC complexes^36^
ma-dm-hisrep	268	Structural prediction screen for histone complexes^37^
ma-osf-ppp2r2a	273	PP2A-B55 protein phosphatase design^38^
ma-denv	31	Dengue virus proteins

## Appendix A. Supplementary material

Supplementary material to this article can be found online at https://doi.org/10.1016/j.jmb.2025.168996.
