# Supplementary material for: ModelArchive: A Deposition Database for Computational Macromolecular Structural Models
Source: J Mol Biol. Author manuscript; Available in PMC 2026 Jul 15. (PMC13370677; doi:10.1016/j.jmb.2025.168996)
Supplement: 1 [file NIHMS2171745-supplement-1.pdf]

ModelArchive: a deposition database for computational macromolecular structural models

Gerardo Tauriello, Andrew M. Waterhouse, Juergen Haas, Dario Behringer, Stefan Bienert, Thomas Garello, Torsten Schwede

SUPPLEMENTARY MATERIAL

ModelArchive web service

Architecture

The current deployment of the ModelArchive consists primarily of a Unix file system containing 1,644,559 files (360GB, compressed). These files contain coordinates, images, metadata and user-uploaded associated data. Although it contains over 600,000 models, the size of the archive is still very manageable and is expected to scale well, although model size requirements vary widely and it is difficult to extrapolate from the current archive to future requirements. There are also ongoing efforts to move the data collection and archiving to a system compatible with PDB-IHM, which will run in the cloud and give us more flexibility to scale up as needed. To make the data accessible and searchable, a PostgreSQL database stores minimal information about the workflow status of each entry. Indexed fields include private access, public release and DOI registration dates, as well as a PostgreSQL search vector field. This database is currently 67MB in compressed export format.

The Django Rest Framework (DRF) is used to serve metadata and coordinates from the file system to users. DRF handles authentication and permissions, especially when accessing unpublished entries. Its class-based views simplify the implementation of POST, GET and DELETE requests during the curation process of an entry. All actions and responses are managed through this REST API, with input and output in JSON format. Although not intended for direct use by external users or advertised as an OpenAPI schema, the API can be accessed programmatically by users if desired.

The frontend for ModelArchive is implemented using React, a JavaScript library for building user interfaces. Previously, AngularJS was used to build the frontend, but React was favoured due to its relative simplicity, JSX-based functional component architecture, and improved performance with virtual DOM rendering.

Nginx serves as the web server in front of Django. It acts as a high-performance reverse proxy server and load balancer for ModelArchive, serving the static images, coordinate files, CSS, JavaScript and HTML that make up the website.

The web server has modest hardware requirements and is currently running on a virtual machine with 16GB of memory and two virtual CPUs running at 2.2GHz.

Data import

Currently, users can upload individual coordinate files in PDB or mmCIF format as the first step in the deposition process. Files uploaded in PDB format are converted to mmCIF format using MAXIT (https://sw-tools.rcsb.org/apps/MAXIT/). ModelCIF formatted coordinates are

imported directly into the ModelArchive file system and indexed in the PostgreSQL database. The Gemmi library (https://gemmi.readthedocs.io) is used during the import of ModelCIF files to extract specific fields such as title, authors, software, and entity information, which are stored in JSON format for quick parsing later by the DRF.

For model sets where multiple models are grouped under a single accession code, users will need to contact the ModelArchive team to coordinate the placement of the files. This is still an ad hoc solution depending on the size and scope of the project and will be replaced in the future as we are currently working on a different data harvesting system that will be compatible with PDB-IHM and will simplify the generation of ModelCIF files for depositors. For current depositions, we are working with the depositors to collect additional data to populate the metadata fields of the ModelCIF format and to define the type of validation to be performed on the data. Conversion to ModelCIF is facilitated by Ben Webb's Python ModelCIF package (https://pypi.org/project/modelcif/). The Python scripts used for most previous conversions are made available at https://git.scicore.unibas.ch/schwede/modelcif-converters. The various projects in the git repository contain enough use cases so that new depositions can be handled by modifying existing scripts rather than building new pipelines from scratch. The final step in any model set conversion is to validate the resulting ModelCIF files. Our pipeline (https://git.scicore.unibas.ch/schwede/modelcif-converters/tree/main/validation) wraps a Gemmi-based Python script around RCSB's CifCheck tool (https://github.com/rcsb/cpp-dict-pack). This tool is available as a containerised solution (https://git.scicore.unibas.ch/schwede/modelcif-converters/container_registry), which facilitates format and data consistency checking.

Data export

When ModelCIF files containing sequences referencing UniProtKB are imported into ModelArchive, they can be indexed, making them discoverable through ModelArchive's text search and external resources such as the 3D-Beacons API. This API allows services such as the SWISS-MODEL Repository, PDBe-KB and applications such as Jalview to retrieve metadata and coordinates using UniProt identifiers.

In addition to the 3D-Beacons API, we can also create custom exports of the data for use in other external resources. For example, for ViralZone to associate viral proteins with available models in ModelArchive, it was sufficient to generate a CSV file mapping UniProt identifiers to ModelArchive identifiers. The data export for Foldseek, on the other hand, includes all models in ModelArchive with summary information including release date and model quality estimates in JSON format and an additional tar.gz archive containing all model coordinates.

Most model sets include downloadable compressed ZIP archives containing all the coordinate files in the dataset. For example, the “Dataset” section of https://modelarchive.org/doi/10.5452/ma-bak-cepc contains a link to download “dataset.zip”. The associated data for the entries is not included in these archives to keep the size manageable, but the location of the associated data can be accessed programmatically in the ModelCIF formatted coordinate files. For very large model sets, the ZIP archive is only created on user request.

Supplementary Figures

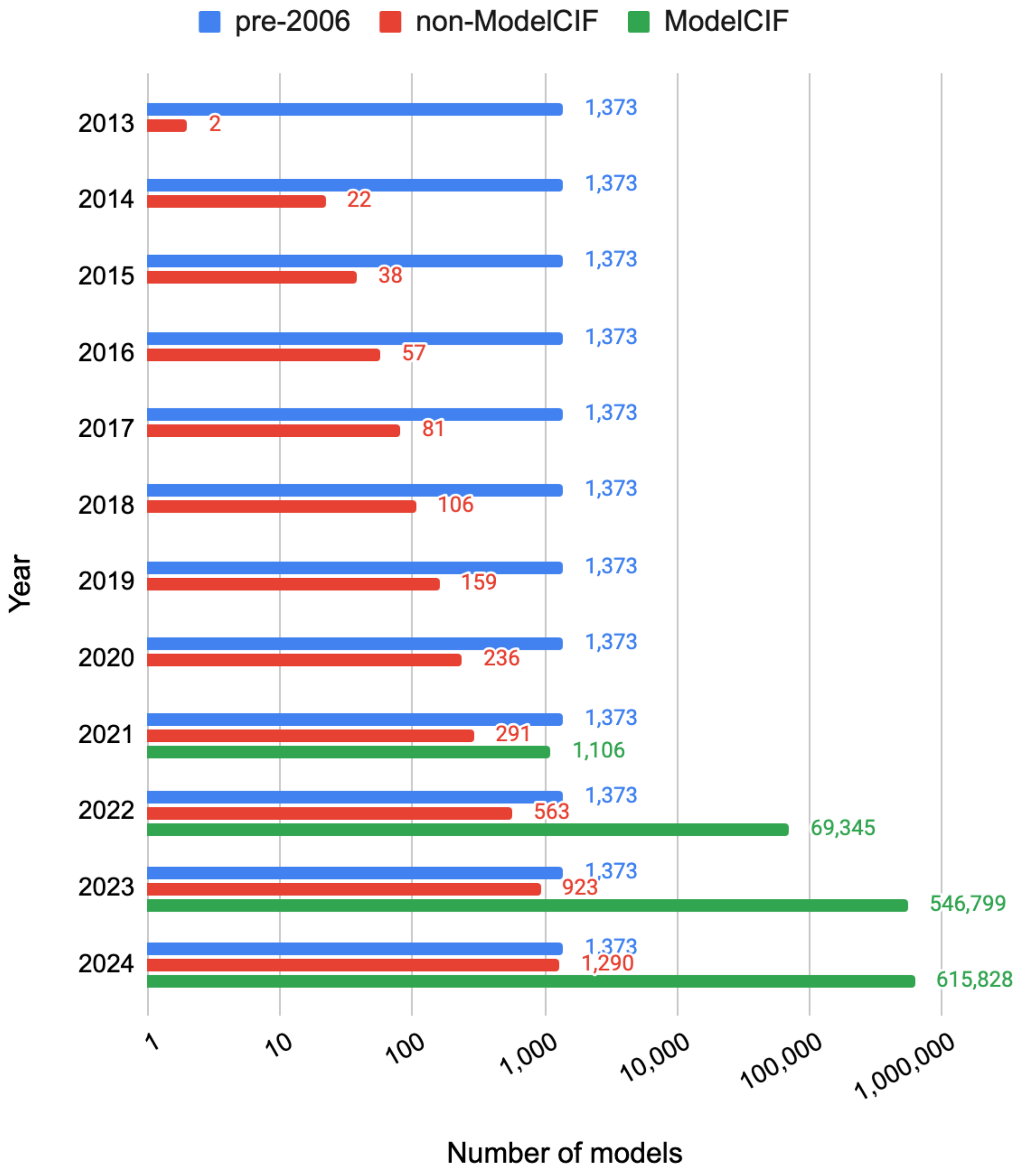

Suppl. Figure 1. Historical growth of entries since ModelArchive started accepting depositions in 2013, shown on a log scale. The archive started with 1,373 legacy models migrated from the PDB and published between 1978 and 2006. The vast majority of models (615,828) are grouped into model sets (see Table 1 in the main text) using the ModelCIF format and have been added in the last four years. The remaining models (1,290) are contained in individual non-ModelCIF entries with free-text metadata descriptions.
